# Musculoskeletal Rehabilitation: New Perspectives in Postoperative Care Following Total Knee Arthroplasty Using an External Motion Sensor and a Smartphone Application for Remote Monitoring

**DOI:** 10.3390/jcm12227163

**Published:** 2023-11-18

**Authors:** Mirjam Victoria Neumann-Langen, Björn Gunnar Ochs, Jörg Lützner, Anne Postler, Julia Kirschberg, Khosrow Sehat, Marius Selig, Thomas M. Grupp

**Affiliations:** 1 Klinikum Konstanz, Department of Orthopaedic and Trauma Surgery, Mainaustrasse 35, 78464 Konstanz, Germany; gunnar.ochs@glkn.de; 2 University Center of Orthopaedic, Trauma and Plastic Surgery, University Hospital Carl Gustav Carus Dresden, TU Dresden, Fetscherstrasse 74, 01307 Dresden, Germany; joerg.luetzner@uniklinikum-dresden.de (J.L.); anne.postler@uniklinikum-dresden.de (A.P.); 3 Waldkliniken Eisenberg GmbH, Klosterlausnitzer Strasse 81, 07607 Eisenberg, Germany; j.kirschberg@waldkliniken-eisenberg.de; 4 Department of Trauma and Orthopaedics, Nottingham University Hospitals NHS Trust, Nottingham NG7 2UH, UK; khosrow.sehat@nuh.nhs.uk; 5 Aesculap AG Research and Development and Medical Scientific Affairs, Am Aesculap-Platz, 78532 Tuttlingen, Germany; marius.selig@aesculap.de (M.S.); thomas.grupp@aesculap.de (T.M.G.); 6 Department of Orthopaedic and Trauma Surgery, Musculoskeletal University Center Munich (MUM), LMULudwigs Maximilian University, 81377 Munich, Germany

**Keywords:** musculoskeletal disease, rehabilitation, osteoarthritis, total knee arthroplasty, external movement sensor, smartphone application

## Abstract

Background: The number of total knee replacements performed annually is steadily increasing. Parallel options for postoperative care are decreasing, which reduces patient satisfaction. External devices to support physical rehabilitation and health monitoring will improve patient satisfaction and postoperative care. Methods: In a prospective, international multicenter study, patients were asked to use an external motion sensor and a smartphone application during the postoperative course of primary total knee arthroplasty. The collected data were transferred to a data platform, allowing for the real-time evaluation of patient data. Results: In three participating centers, 98 patients were included. The general acceptance of using the sensor and app was high, with an overall compliance in study participation rate of up to 76%. The early results showed a significant improvement in the overall quality of life (*p* < 0.001) and significant reductions in pain (*p* < 0.01) and depression (*p* < 0.001). Conclusions: The early results of this clinical and multicenter study emphasize that there is a high interest in and acceptance of digital solutions in patients’ treatment pathways. Motion sensor and smartphone applications support patients in early rehabilitation.

## 1. Introduction

Musculoskeletal diseases, such as osteoarthritis, progressively affect medical healthcare systems. The impact of osteoarthritis is described as 10% in men and 13% in women, especially for symptomatic knee osteoarthritis [1]. It causes a significant economic burden to healthcare systems, with USD 41.7 billion spent in 2013 for hospitalization charges on knee replacements [2]. A systematic review published in 2023 puts the current average costs of TKA surgery at USD 15,616 per patient [3]. Total knee arthroplasty (TKA) is proven to be cost-effective and to improve quality of life [4]. Most patients following TKA are satisfied, but up to 20% of treated patients remain unsatisfied following TKA due to unmet expectations, residual pain, and postoperative complications [5,6,7]. In addition to intrinsic factors such as surgical or patient-related factors, the fulfilling of patient expectations has been linked with postoperative satisfaction [8,9]. The analysis of these unfulfilled expectations should be focused on assistive mobile health (m-health) devices.

In general, osteoarthritis (OA) leads to immobility, disability in daily routines, and loss of quality-of-life, often resulting in chronic pain [10]. Managing OA is a multidisciplinary approach involving different disciplines, including pain management, physiotherapy, improvement or amendment of housing and living standards, surgical intervention (e.g., total joint replacement), and postoperative aftercare (e.g., rehabilitation facilities). The demographic change towards an aging population requires more physical human support workers, such as rehabilitation care managers or physiotherapist. However, the numbers of trainees are decreasing, instead. A total of 1901.585 physiotherapists are currently practicing worldwide, but in 2019, the Lancet stated that 2.41 billion individuals had conditions that would benefit from rehabilitation [11]. The awareness of this background information has raised interest in developing wearable equipment, sensors, and connecting technologies to monitor vital signs, physical activities, and fitness. The main goal of these devices is to increase the autonomy of patients and improve their independence from stationary rehabilitation centers while still allowing for individual, continuous, and supervised rehabilitation. The convenient and easy access to technology enables the continuous monitoring of individuals [12]. This, in turn, enables the analysis of bioelectrical signals and movements, which can aid in the diagnosis, prevention, and analysis of a wide range of main medical concerns. Remote and ambulatory monitoring are growing needs in healthcare systems [13]. Moreover, remote monitoring allows access to an increased data volume and, therefore, can promote a better diagnosis for diseases [14], improve the individual’s sports performance [15,16], and accelerate patient rehabilitation processes [17,18].

The purpose of this multicenter, international clinical pilot study was to analyze real-time patient use of an external motion sensor, a smartphone application, and its connectivity to a data platform (Pheno4U, Aesculap AG, Tuttlingen, Germany) following primary total knee arthroplasty. The clinical results will identify the relevant challenges in musculoskeletal rehabilitation phases to improve remote monitoring and external sensor device use. The overall ability for collecting treatment pathway and patient-related data will enable complication predictions and more individual treatment schemes.

## 2. Materials and Methods

**Figure 1:**
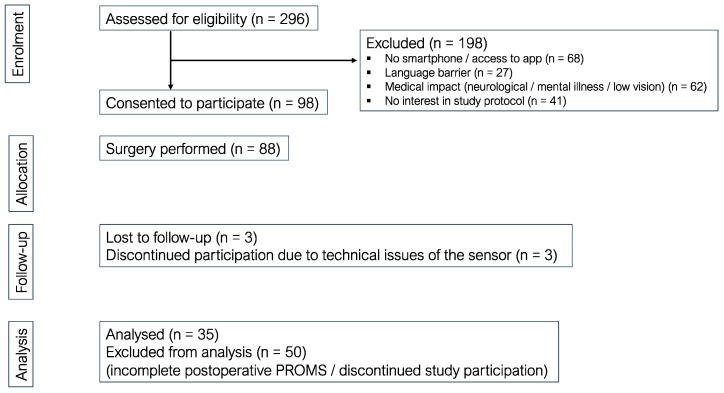
Flow chart describing the eligibility of included patients and exclusion criteria.

The study design is a prospective, non-interventional, multinational clinical pilot study conducted at six sites in Germany, France, and the UK with a sample size of 600 patients. The objectives are data collection of the complete treatment protocol during TKA surgery and rehabilitation for each patient using the Pheno4U data platform (Pheno4U, Aesculap AG, Tuttlingen, Germany). No specific primary endpoint is defined due to the prospective nature of this study. The goal of the data platform is to link data from the hospital information system (HIS), i.e., the patient data collected and stored in a mobile application on the individual patient’s smartphone called ForPatientApp (FPA), and the use of devices (motion sensor) throughout the treatment pathway. Patients are asked to answer questionnaires (PROMS) using the smartphone app about their health status pre-operatively, and at 2 weeks, 6 weeks, 6 months, and 9 months postoperatively. Furthermore, patients are asked to use an external motion sensor and a smartphone app to measure the active range of motion (ROM). Patients scheduled for a primary total knee arthroplasty were assessed for eligibility. Patients were approached during consultation for participation in this study. All study sites have high surgical experience, with up to 500 primary TKA implantations per year. Inclusion criteria were primary total knee arthroplasty, owning and using a mobile phone, native language skills, and overall general interest in participating in an observational study (Figure 1).

Before enrolling in this study, all participating patients were informed about the study content, the course of this study, any risks, the duration of participation, and the voluntary nature of study participation. The participating patients each signed an informed consent form.

### 2.1. Hospital Information System

The hospital information systems were inhomogeneous due to the international nature of this study. In general, demographic data regarding patients’ age, gender, height, and body weight for the analysis of body mass indexes (BMIs) were collected. The preoperative status, including chronic diseases like hypertension, diabetes mellitus, or rheumatoid arthritis, and the daily need for medication were documented. Environmental or medical allergies and chronic viral diseases like HIV or Hepatitis were inquired and documented. All patient-related information was summarized and transferred pseudo-anonymously to the Pheno4U server.

### 2.2. Electronic Application System (App)

The electronic application system (FPA) was developed and created by Aesculap AG and was implemented on the smartphones of the participating patients. To allow data analysis, a numerical Unique Case Identifier (UCID) was generated for each patient. Exercises for individual training sessions at each post-operative phase were embedded within the FPA app. Furthermore, PROMS were included, which the patients were asked to complete during different stages of the rehabilitation process.

### 2.3. External Motion Sensor

An external motion sensor was placed just below the knee joint to detect and store the range-of-motion of the affected knee joint. The sensor device was worn during the movement exercises and aligned in space using a rotation sensor. The validity of the motion sensor has been proven in a laboratory setting with healthy adults [19]

### 2.4. Patient-Reported Outcome Measures (PROMS)

**Figure 2:**
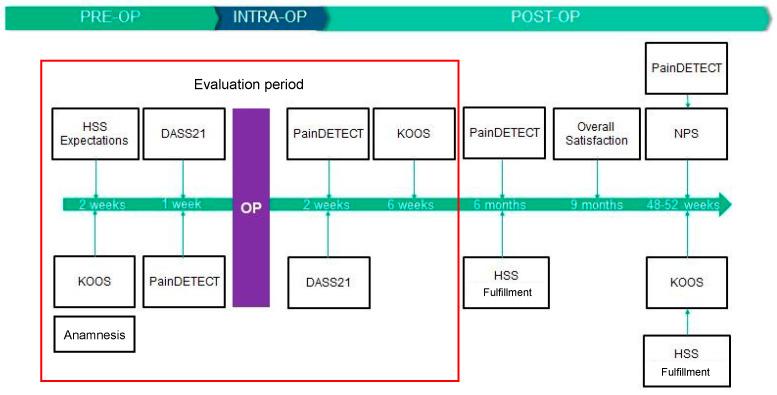
Timeline of the questionnaires collected during the pre- and postoperative period. The red box marks the period of questionnaires evaluated so far.

The participating patients were asked to complete various surveys during this study: the KOOS [20], HSS Knee Replacement Expectation Survey [21,22], Pain (Pain DETECT) [23], and the DASS 21 [24]. For an objective assessment of the external motion sensor and the mobile application, the Net Promoter Score was obtained, which showed participant satisfaction and retention [25]. The PROMS were entered directly to the FPA app at 2 weeks, 6 weeks, 6 months, and 9 months postoperatively (Figure 2). The subjective data collected with the surveys can be compared with the functional movement data and correlated with possible influences such as medical risk factors.

### 2.5. Data Platform (Pheno4U)

**Figure 3:**
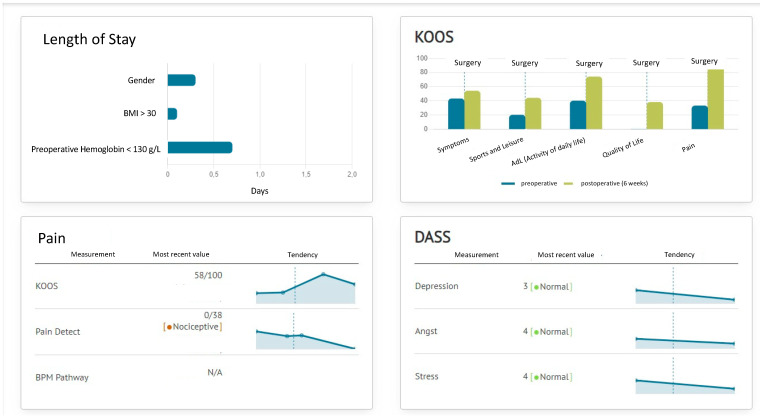
Screenshot of the dashboard with presentation of selected dates of interest.

Within the data platform, the individual patients’ data sets were matched to functional outcome, data collected from the external motion sensor device, and subjective outcome data collected from the ForPatientApp. The dynamic and objective progress of the functional improvement in the postoperative ROM, as well as the subjective improvement in pain relief and increasing mobility, are presented in scales and tables (Figure 3). The real-time data and analyses for individual patients and groups of patients were displayed in a dedicated dashboard, allowing for the identification of potential risk factors.

### 2.6. Data Analysis

All results were expressed at means ± standard deviation. Statistical analysis was performed using the R 4.2.2 software. A paired *t*-test was used to compare the preoperative and postoperative scores. Significance was accepted at *p* < 0.05.

The study protocol was primarily approved by the local Human Research Ethics Committee of the Technical University of Dresden, Germany (ethical approval code: BO-EK-123032022), and by the local ethics committees of the participating centers. This study was registered at clinialtrials.gov under NCT05302934.

## 3. Results

Ninety-eight patients from three out of four participating study centers participated in this study. The general acceptance of using a motion sensor and the FPA App and participation in this study was high, with an overall compliance rate of up to 76%. Compliance was defined as using the motion sensor at least once a week and fulfilling the PROMs. The lack of smartphone accessibility was a primary reason for not participating, followed by medical impacts.

There were 49 male and 43 female participants, with an average age of 63 years (41–80 years). An overview of the patients’ demographics is given in Table 1.

The indication for primary total knee arthroplasty was varus (43%, *n* = 43) or valgus (3%, *n* = 3) osteoarthritis of the knee joint or general osteoarthritis in 53 patients (54%). All patients underwent navigated surgery with either general or spinal anesthesia.

### 3.1. Functional Results

During the first six weeks, there was an improvement in the range of motion (ROM), with a median of 90/5/0° for flexion and extension. Within the first 3 months, patients demonstrated further improvements in flexion parameters of up to 120°.

### 3.2. Patient-Reported Outcomes

**Figure 4:**
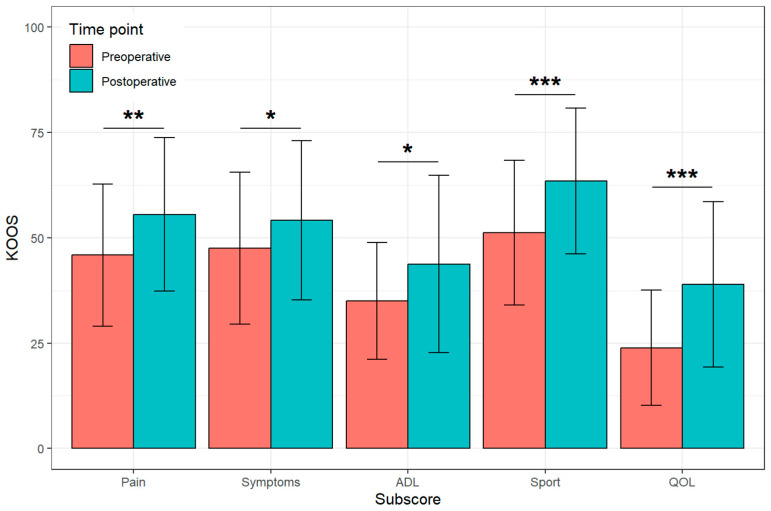
Of the key points recorded by the KOOS score, there was a significant improvement in pain, symptoms, activity in daily life (ADL), sport, and quality of life (QOL) [* *p* < 0.05, ** *p* < 0.01, *** *p* < 0.001].

Of the patients included thus far, 54 patients completed the KOOS questionnaire pre- and postoperatively at 6 weeks. A total of 45 patients completed the DASS questionnaire pre- and postoperatively at 2 weeks. The KOOS analysis showed that pain (*p* < 0.01), clinical symptoms like swelling (*p* < 0.05), general activity in daily life (*p* < 0.05), sport abilities (*p* < 0.001), and overall quality of life (*p* < 0.001) significantly improved (Figure 4).

**Figure 5:**
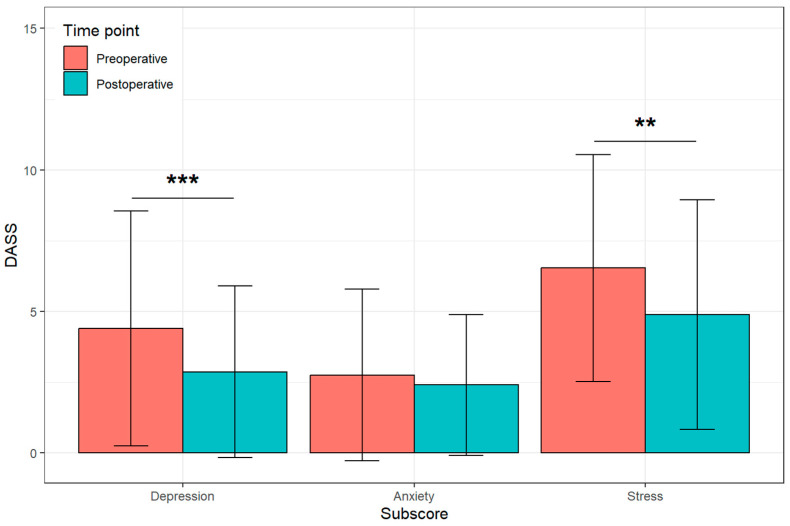
Presentation of the different DASS classifications with significant postoperative improvements using the smartphone app and the external movement sensor [** *p* < 0.01, *** *p* < 0.001].

The detected DASS 21 score significantly improved from pre- to postoperative values for stress (*p* < 0.01) and depression (*p* < 0.001) (Figure 5).

The HSS expectation score was 57.6 ± 7.5 (34–68), calculated from 87 patients. The HSS fulfillment score was not part of the early postoperative evaluation at this stage.

## 4. Discussion

The results of this international, multicenter clinical pilot study showed that there is high acceptance of using external motion sensors and smartphone application technology and participating in a clinical, voluntary study. Only three patients were lost to follow-up, due to technical problems with the motion sensor. No patient declined or refused to further participate in this study. In total, 68 out of 97 patients completed at least one PROM during follow-up, which allowed for a very early but subjective analysis of the postoperative outcome. Overall, pain relief and improved quality of life, including daily activity, significantly improved in the very early postoperative course following primary total knee arthroplasty, with the use of an external motion sensor and smartphone application. Digital support has helped the patients in their clinical and subjective functional outcomes. The patients included in this study were of a younger average age, which meant they were more familiar with mobile devices and had a greater interest in digital applications.

Comparison with other clinical studies focusing on the use of a single motion sensor and additional smartphone applications following primary total knee arthroplasty is limited. Numerous studies have investigated inertial and force sensors for gait analysis, knee angle, and foot plantar pressure during gait [26,27,28]. Their purpose is to improve walking abilities in elderly, stroke, multiple sclerosis, and Parkinson’s disease patients. In a Chinese clinical study, 21 post-stroke patients participated in a gait analysis using three different modal models of marker trajectory, ground reaction force, and electromyogram. Data from these three modalities were simultaneously collected during gait analysis and compared to a healthy volunteer group [29]. The recognition and estimation performance reached 98% accuracy when using a multimodal integration data set [29].

Another clinical study observed 17 physiotherapy patients using an inertial measurement unit (IMU) and surface electromyography (sEMG) sensors to track exercise [30]. The authors obtained an average accuracy of 96% in detecting issues such as acceleration, rotation, angular velocity, and posture information in the monitored exercises. For upper limb training, sEMG sensors and accelerometers were used in an overall verification for the feasibility and usability of the system and a wearable application associated with game-based training and user feedback systems, showing that three of twenty participating subjects could improve their game performance through repetitive training [31].

A portable vestibular rehabilitation device was investigated in 10 subjects to train at home as part of a rehabilitation program [32]. The device consists of a head and a base unit and could increase the vestibulo-ocular reflex (VOR) by 11%.

A French group presented a system using inertial sensors and a Kinect Camera implemented in a game scenario to improve upper and lower limb rehabilitation following a stroke in 2018. Eight post-stroke patients participated and filled in questionnaires. The results showed that multi-sensor fusion provides useful data for clinical follow-up, and the game scenarios led to high immersion in the participating subjects [33].

Recently, the number of m-health devices has increased, whether their purpose is for fitness, health, or medical reasons. The financial development of this mobile health market has skyrocketed between the years of 2017 and 2019, from USD 6.95 to 25.17 billion just in the US [34,35]. These numbers should mirror the overall interest and awareness in developing user-friendly devices. The challenge of this market is to combine m-health devices for general functions and ease of use. The advantages of the devices are obvious, but there are many challenges in determining effectiveness. There are concerns about validation, standardization, and interoperability [36]. Other issues include data security, cost effectiveness, and the reusability of the wearable sensors. Moreover, most m-health devices are developed for a generation that is used to mobile devices and smartphone applications rather than elderly adults in postoperative care. Evidence for the efficacy of m-health devices is proven in many studies. In long-term use studies, the durability, usability, and ease of use must be proven further.

The presented clinical pilot study has some limitations that must be considered carefully. First, the number of participating subjects was small, and the follow-up time was short. Second, the international nature of this study may bias the comparison of patients’ objective data due to individual or national personalities and the burden of rehabilitation possibilities. Third, the presented mobile health devices were developed for patients following primary total knee arthroplasty to improve their postoperative satisfaction. However, the following strengths of this study should be highlighted: to our knowledge, this is the first clinical study that accompanies patients following primary total knee arthroplasty with an external motion sensor and a smartphone application summarizing real-time data on a platform with immediate data interpretation and intervention in complicated postoperative courses. Although the evaluated data presented are limited, the preliminary results are promising in that therapy supported by external sensors and mobile smartphone applications with conventional rehabilitation measures leads to earlier activity and higher patient satisfaction. Another approach to using external motion sensors could be by implementing them in the preoperative rehabilitation course for muscle strengthening to improve postoperative outcomes.

## 5. Conclusions

The use of an external movement sensor and smartphone application supports patients following primary total knee arthroplasty in their early functional and objective results. The evaluation of the long-term results is pending, and definitive statements cannot be made about the exact clinical use of this sensor, the smartphone application, and the data platform as yet.

## Figures and Tables

**Table 1:** Tabular overview of patients’ demographics and risk factors.

	Female: 43 (46.7%)	Male: 49 (53.3%)	Total
Age (at surgery) [years]	61.7 ± 7.5 (45–77) (*n* = 36)	63.8 ± 7.8 (41–80) (*n* = 48)	62.9 ± 7.7 (41–80) (*n* = 84)
**Risk factors**			
None	12 (33.3%) (*n* = 36)	16 (33.3%) (*n* = 48)	28 (33.3%) (*n* = 84)
BMI [kg/m²]	30.4 ± 6.3 (19–42) (*n* = 36)	30.0 ± 5.1 (21–46) (*n* = 48)	30.1 ± 5.6 (19–46) (*n* = 84)
Smoking	5 (13.9%) (*n* = 36)	6 (12.5%) (*n* = 48)	11 (13.1%) (*n* = 84)
Diabetes	4 (11.1%) (*n* = 36)	6 (12.5%) (*n* = 48)	10 (11.9%) (*n* = 84)
Hypertension	20 (55.6%) (*n* = 36)	27 (56.3%) (*n* = 48)	47 (56.0%) (*n* = 84)
Total reviewed	*n* = 36	*n* = 48	*n* = 84

## Data Availability

Data are contained within this article.
